# Selection Maintains Protein Interactome Resilience in the Long-Term Evolution Experiment with *Escherichia coli*

**DOI:** 10.1093/gbe/evab074

**Published:** 2021-04-20

**Authors:** Rohan Maddamsetti

**Affiliations:** Department of Biomedical Engineering, Duke University, Durham, North Carolina, USA

**Keywords:** experimental evolution, purifying selection, protein–protein interaction network, evolutionary systems biology

## Abstract

Most cellular functions are carried out by a dynamic network of interacting proteins. An open question is whether the network properties of protein interactomes represent phenotypes under natural selection. One proposal is that protein interactomes have evolved to be resilient, such that they tend to maintain connectivity when proteins are removed from the network. This hypothesis predicts that interactome resilience should be maintained by natural selection during long-term experimental evolution. I tested this prediction by modeling the evolution of protein–protein interaction (PPI) networks in Lenski’s long-term evolution experiment with *Escherichia coli* (LTEE). In this test, I removed proteins affected by nonsense, insertion, deletion, and transposon mutations in evolved LTEE strains, and measured the resilience of the resulting networks. I compared the rate of change of network resilience in each LTEE population to the rate of change of network resilience for corresponding randomized networks. The evolved PPI networks are significantly more resilient than networks in which random proteins have been deleted. Moreover, the evolved networks are generally more resilient than networks in which the random deletion of proteins was restricted to those disrupted in LTEE. These results suggest that evolution in the LTEE has favored PPI networks that are, on average, more resilient than expected from the genetic variation across the evolved strains. My findings therefore support the hypothesis that selection maintains protein interactome resilience over evolutionary time.

**Significance**

Understanding how protein–protein interaction (PPI) networks evolve is a central goal of evolutionary systems biology. One property that has been hypothesized to be important for PPI network evolution is resilience, which means that networks tend to maintain connectivity even after many nodes (proteins in this case) have been removed. This hypothesis predicts that PPI network resilience should be maintained during long-term experimental evolution. Consistent with this prediction, I found that the PPI networks that evolved over 50,000 generations of Lenski’s long-term evolution experiment with *Escherichia coli* are more resilient than expected by chance.

## Introduction

When redundant nodes and connections in a network carry a cost, removing those redundancies may increase efficiency at the expense of reducing resilience to unexpected disruptions. After a critical point, called the percolation threshold, further pruning can cause a catastrophic breakdown of connectivity and function (Callaway et al. 2000). In the context of protein–protein interaction (PPI) networks, efficiency may refer to the cost of protein production (Kafri et al. 2016).

Zitnik et al. (2019) formally defined network resilience to measure how quickly a network breaks down as nodes are removed. They then studied the evolution of PPI networks (also called protein interactomes) across the tree of life, concluding with the hypothesis that interactome resilience is favored during evolution. While interesting, this hypothesis is rather vague. To better understand the relevance of network resilience to evolutionary biology, we need to ask whether network resilience has any relevance or predictive power in additional contexts. If network resilience is a *necessary* property of evolved PPI networks, then it should be maintained by selection during long-term evolution experiments. Here, I use the methods developed by Zitnik et al. (2019) to test this prediction, by examining how protein interactome resilience has evolved in Lenski’s long-term evolution experiment with *Escherichia coli* (LTEE).

In the LTEE, 12 initially identical populations of *E. coli* have evolved for more than 50,000 generations (Lenski et al. 1991; Lenski 2017). The LTEE populations are named by the presence of a neutral phenotypic marker: populations Ara+1 to Ara+6 grow on arabinose, whereas populations Ara−1 to Ara−6 cannot (Lenski et al. 1991). Many populations have lost unnecessary metabolic traits (Leiby and Marx 2014; Grant et al. 2021), and many genes have been disrupted by loss-of-function mutations, in part caused by the evolution of elevated mutation rates in several populations (Tenaillon et al. 2016; Couce et al. 2017; Good et al. 2017; Maddamsetti and Grant 2020a).

Despite evidence for genomic and phenotypic streamlining, it is unknown how PPI network resilience has evolved during the LTEE. To examine this question, I compared the rate of change of protein interactome resilience in each LTEE population to the expected rate of change in corresponding sets of randomized networks (Materials and Methods). I compare rates of change of interactome resilience between real and simulated networks, because this approach is simple and accounts for phylogenetic correlations among genomes from the same population; it treats the independent LTEE populations as the appropriate unit of statistical replication. Importantly, the randomized networks within each population have no such phylogenetic correlations, in order to reduce the computational cost of sampling large numbers of statistically independent replicates. For robustness, I analyzed two curated data sets of PPIs in *E. coli*, which I refer to as Zitnik interactome (Zitnik et al. 2019) and the Cong interactome (Cong et al. 2019). Overall, protein interactome resilience is higher in the LTEE than in the randomized networks, indicating that this system-level property is being maintained over long-term experimental evolution.

## Materials and Methods

The full Materials and Methods section is in the supplementary information, Supplementary Material online; a brief summary follows. For robustness, I conducted two separate analyses, using two PPI data sets (Cong et al. 2019; Zitnik et al. 2019). For each of these PPI data sets, I generated a protein interactome network for the ancestral LTEE clone, REL606. I then generated 264 evolved protein interactome networks, in correspondence with the 264 genomes of LTEE clones isolated at 11 timepoints through 50,000 generations (Tenaillon et al. 2016). To generate the evolved networks, I first tabulated nonsense SNPs, small indels, mobile element insertions, and large deletions affecting protein-coding regions in each genome (Tenaillon et al. 2016). Because these mutations disrupt protein reading frames, I use them as a proxy for loss-of-function mutations in the LTEE. For this reason, I call these types of mutations “gene disruptions,” and call genes that are affected by these types of mutations “disrupted genes” for short. I then constructed the evolved networks by pruning the REL606 interactome of nodes (proteins) and edges (interactions) affected by gene disruptions in the given genome. Network resilience was calculated using the method described in Zitnik et al. (2019). Please see the full Materials and Methods section in the supplementary information, Supplementary Material online for further details about the data sets, the network resilience calculations, and the statistical analysis.

## Results

### LTEE PPI Networks Are More Resilient Than PPI Networks with Random Proteins Deleted

**Fig. 1.**
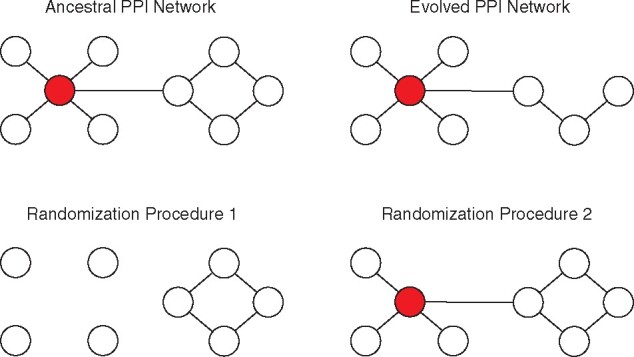
Illustration of ancestral, evolved, and randomized networks. Nodes represent proteins, and edges represent protein–protein interactions. Red nodes indicate essential proteins which cause lethal phenotypes if disrupted by nonsense SNPs, small indels, mobile element insertions, or large deletions. The PPI network of the ancestral bacterial clone is shown at top left. The PPI network of an evolved bacterial clone, in which one protein has been disrupted, is shown at top right. The randomization procedures sample subnetworks of the ancestral PPI network. Each randomized network corresponds to an evolved PPI network: the number of proteins disrupted in the randomized network is fixed to the number disrupted in the corresponding evolved PPI network. The first randomization procedure used in this article (bottom left) samples all protein-coding genes in the ancestral clone for disruption, including those encoding essential proteins. The second randomization procedure used in this article (bottom right) samples protein-coding genes for disruption based on the number of evolved populations that contain disruptions of that gene (see Materials and Methods for further details).

**Fig. 2.**
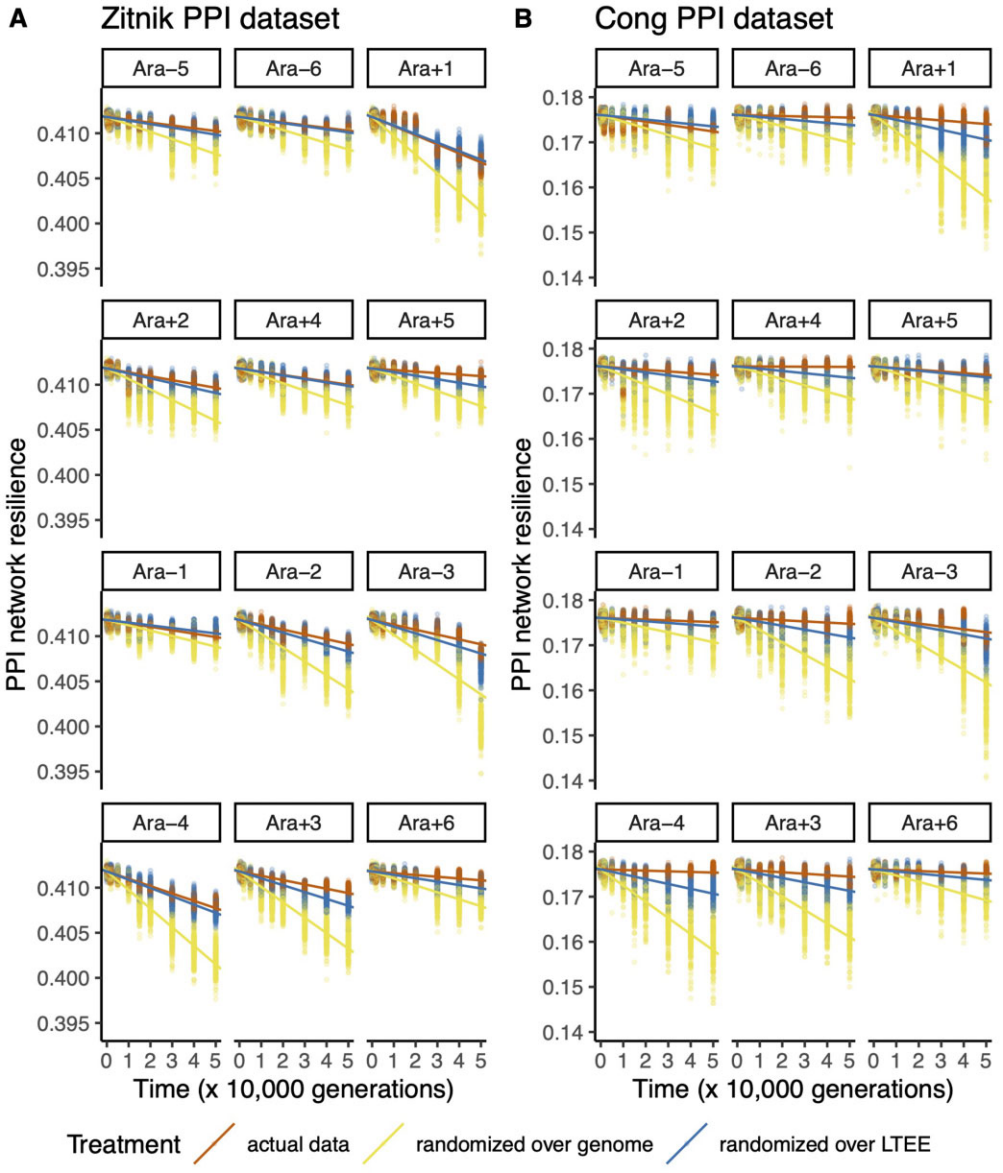
Protein–protein interaction (PPI) networks in the LTEE (red) lose network resilience more slowly than randomized networks generated using either all genes in the REL606 genome (yellow) or only those disrupted in the LTEE (blue). The top six populations have the ancestral point-mutation rate, whereas the bottom six populations evolved elevated point-mutation rates. (*A*) Analysis based on the *Escherichia coli* PPI network published in Zitnik et al. (2019). (*B*) Analysis based on the *E. coli* PPI network published in Cong et al. (2019).

I calculated the resilience of randomized counterparts of the evolved LTEE PPI networks, in which proteins to delete from the network were sampled at random. In this randomization scheme, a protein may be removed from the PPI network, regardless of its essentiality in *E. coli* (fig. 1). For this reason, I expected that the evolved LTEE PPI networks would be more resilient than the randomized PPI networks, and this was indeed the case (Zitnik interactome: Wilcoxon signed-rank exact test *P *=* *0.00024; Cong interactome: Wilcoxon signed-rank exact test *P *=* *0.00024). This result can be seen by comparing the red and yellow slopes in figure 2.

The analysis in figure 2 also shows that the Ara+1 population is an outlier: randomized Ara+1 networks are expected to have very low resilience, even though the Ara+1 population has maintained the wild-type point mutation rate (see fig. 2 legend). This finding can be explained by the high transposon insertion mutation rate that evolved in this population (Papadopoulos et al. 1999; Maddamsetti and Grant 2020a; Consuegra et al. 2021). The Ara+1 population has also lagged behind the others in mean fitness (Wiser et al. 2013; Grant et al. 2021; Consuegra et al. 2021).

### LTEE PPI Networks Are More Resilient Than PPI Networks with Gene Disruptions Sampled across LTEE Populations

In part, the previous finding could be caused by sampling unrealistic randomized networks. For instance, the previous method permits the sampling of randomized networks that lack essential ribosomal proteins, which is biologically implausible (fig. 1). I therefore conducted a second test, in which I restricted the proteins sampled for deletion to those that were disrupted in at least one LTEE population. Here, the probability of sampling proteins for deletion was weighted by the frequency of observed disruptions across LTEE populations. This resampling procedure takes parallel genetic evolution into account (Woods et al. 2006; Ostrowski et al. 2008; Tenaillon et al. 2016), because proteins that are disrupted multiple times across populations are more likely to be sampled. The evolved LTEE PPI networks, on the whole, are more resilient than randomized PPI networks generated from gene disruptions tabulated across all 12 LTEE populations (Zitnik interactome: Wilcoxon signed-rank exact test *P *=* *0.0061; Cong interactome: Wilcoxon signed-rank exact test *P *=* *0.0012). This result can be seen by comparing the red and blue slopes in figure 2.

### Genes in Large Deletions in the LTEE neither Show Physical Modularity nor Fewer Interactions

The resampling procedures operate on the level of individual gene disruptions and losses, such that large deletions are replaced by a sample of individual gene disruptions. Therefore, the resampling procedures could bias the results by breaking up the block structure of multi-gene deletions. This would matter if disrupting a block of *x* genes has less of an effect on PPI network resilience than disrupting *x* genes across the genome.

I examined two PPI properties through which the absence of large deletions in the randomized networks could have an effect. First, systematic bias could be introduced if genes within large deletions tend to have fewer interactions than genes affected by small indels or nonsense mutations. Second, systematic bias could be introduced if interactions within large deletions show physical modularity, such that genes within large deletions preferentially interact with each other, but not with genes elsewhere on the chromosome.

I find no difference in PPI degree between genes knocked out by multi-gene deletions, and those disrupted by single-gene mutations in the 50,000 generation LTEE clones (Zitnik interactome: Wilcoxon rank-sum test *P *=* *0.46; Cong interactome: Wilcoxon rank-sum test *P *=* *0.26). Furthermore, I find that interactions removed by multi-gene deletions in 50,000 generation LTEE clones are *further apart* in the genome, on average, than interactions removed by single-gene disruption mutations. For the case of the Zitnik interactome, interactions removed by multi-gene deletions have a mean genomic distance of 1,071,063 base-pairs, compared with a mean genomic distance of 1,054,403 base-pairs for interactions removed by single-gene disruptions (Wilcoxon rank-sum test: *P *=* *0.0364). For the case of the Cong interactome, interactions removed by multi-gene deletions have a mean genomic distance of 714,316 base-pairs, compared with a mean genomic distance of 583,624 base-pairs for interactions removed by single-gene disruptions (Wilcoxon rank-sum test: *P *<* *10^−4^). Therefore, the differences between realized and randomized PPI network resilience in the LTEE do not seem to be an artifact of the resampling procedure, at least with regard to PPI degree and the aspects of physical modularity that I examined.

### Purifying Selection on Essential Genes in the LTEE Ancestral Clone Is Insufficient to Explain the Maintenance of Network Resilience in the LTEE

**Fig. 3.**
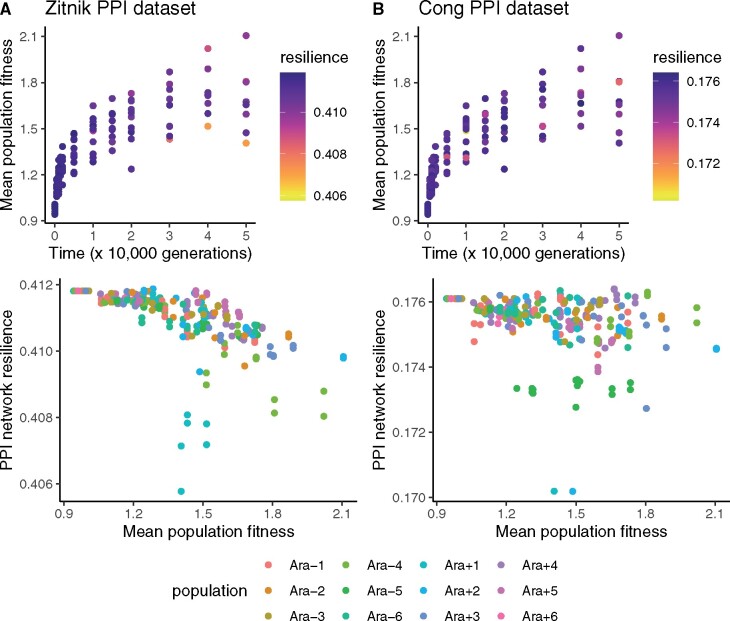
PPI network resilience negatively correlates with mean population fitness, measured by direct competition assays against reference LTEE clones (Wiser et al. 2013). The top panels show the fitness measurements from Wiser et al. (2013); the colors denote the PPI network resilience of genomes sampled from the corresponding populations and time points. The bottom panels show the negative correlations between mean population fitness and PPI network resilience. (*A*) Analysis based on the *Escherichia coli* PPI network published in Zitnik et al. (2019). The negative correlation between fitness and resilience is significant (Pearson’s product-moment correlation: *r* = −0.59, *P < *10^−16^). (*B*) Analysis based on the *E. coli* PPI network published in Cong et al. (2019). The negative correlation between fitness and resilience is significant (Pearson’s product-moment correlation: *r* = −0.27, *P < *10^−4^).

What evolutionary forces are responsible for maintaining protein interactome resilience in the LTEE? Interactome resilience is not a target of positive selection, because mean population fitness increases in each LTEE population (Wiser et al. 2013) whereas interactome resilience decreases (fig. 2). Therefore, interactome resilience negatively correlates with fitness gains in the LTEE (fig. 3). The only remaining explanation is that network resilience is being maintained by purifying selection. Still, it is unclear whether protein interactome resilience is under direct selection, or whether its maintenance is a byproduct of purifying selection on correlated phenotypes.

As essential genes cause lethal phenotypes when disrupted, direct purifying selection against the disruption of essential genes could cause indirect purifying selection on interactome resilience. I hypothesized that disruptions of essential genes would have disproportionately negative effects on network resilience. Although I found evidence of direct purifying selection on essential genes, I found limited evidence for the hypothesis that direct purifying selection on essential genes indirectly maintains interactome resilience.

I examined a set of 541 essential and nearly essential genes that were identified in REL606 (Couce et al. 2017). These genes are highly enriched for PPIs compared with the remaining 3,571 nonessential genes (one-sided Wilcoxon rank-sum test: *P < *10^−58^ for Zitnik PPI data set, *P < *10^−10^ for Cong PPI data set). Essential and nearly essential genes also show a significant signal of purifying selection in the 50,000 generation LTEE clones: they contain 44 out of 941 gene disruptions in the 50,000-generation LTEE genomes, whereas the total length of essential and nearly essential genes is 499,180 bp, out of 3,962,143 bp representing the total length of all protein-coding genes (one-sided binomial test: *P < *10^−15^). It should be noted that the 23 of the 57 genes with clear evidence of parallel evolution (i.e., two or more nonsynonymous mutations) in nonmutator lineages of the LTEE (Tenaillon et al. 2016; Maddamsetti et al. 2017) are essential or nearly essential. This association between essentiality and positive selection in the LTEE is highly significant (Fisher’s exact test: *P < *10^−6^).

I then simulated the disruption of every single gene in REL606 to measure their effects on network resilience. In contrast with my initial expectation, I find that the majority of single gene disruptions in REL606 *increase* network resilience. Furthermore, disruptions of essential and nonessential genes have qualitatively similar effects on network resilience (supplementary fig. S1, Supplementary Material online). When I examine the effect of disrupting every single gene in the 50,000 generation LTEE clones on interactome resilience, the results are largely strain-specific. Again, the trend for essential genes is similar to the trend for nonessential genes (supplementary fig. S2, Supplementary Material online). Together, these findings suggest that purifying selection on essential genes in the LTEE ancestral clone is not sufficient to explain the maintenance of network resilience in the LTEE.

## Discussion

I find that evolved networks in the LTEE lose protein interactome resilience more slowly than expected, based on comparisons with networks with random gene disruptions and networks with gene disruptions weighted by their occurrence across LTEE populations (fig. 1). The second analysis controls for the biologically implausible PPI networks that would be created by sampling essential genes for disruption. Together, these results are consistent with Zitnik and colleagues’ general hypotheses that network resilience 1) is a genuine property of evolved PPI networks and 2) is relevant for understanding how PPI networks evolve.

Selection must be driving the maintenance of protein interactome resilience in the LTEE. Since protein interactome resilience negatively correlates with fitness gains in the LTEE (fig. 3), positive selection can be ruled out as a cause. Therefore, purifying selection must be maintaining protein interactome resilience in the LTEE. In addition, I find a general trend that loss-of-function mutations *increase* network resilience in REL606. This finding shows that positive selection has not optimized the interactome resilience of REL606: indeed, the interactome resilience of REL606 appears to be closer to a local minimum than to a local maximum.

Protein interactome resilience could be maintained by direct selection, or as a byproduct of selection on phenotypes that correlate with interactome resilience. In previous work, I found evidence for purifying selection on essential genes in metagenomic time series covering 60,000 generations of the LTEE (Maddamsetti and Grant 2020b), as well as evidence of purifying selection on highly interacting genes (Maddamsetti 2021). Consistent with those findings, the LTEE genomes analyzed here show evidence of purifying selection on essential genes, which are also highly enriched for PPIs. I then asked whether purifying selection on essential genes could explain the maintenance of interactome resilience in the LTEE. My analyses suggest that this is not the case: disruptions of essential genes are qualitatively similar to disruptions of nonessential genes, in regard to their effects on network resilience (supplementary figs. S1 and S2, Supplementary Material online). These findings still leave open the broader question of whether protein interactome resilience is under direct selection, or is a byproduct of selection on other, unknown, correlated phenotypes. In this vein, it would be interesting to ask whether variation in interactome resilience correlates with evolvability in the sense of the distribution of fitness effects (DFE) for beneficial mutations (Mustonen and Lässig 2010; Woods et al. 2011; Łuksza and Lässig 2014; Levy et al. 2015; Ba et al. 2019), or with mutational robustness in the sense of the DFE for deleterious mutations (Johnson et al. 2019).

This work has important limitations. First, the resampling procedure used to generate randomized networks does not maintain the block structure of large deletion mutations. For this reason, I analyzed the protein interaction degree and genomic interaction distance distribution of genes affected by large deletions in the LTEE. This analysis did not uncover any systematic biases that would affect the broad import of my findings. Second, the resampling procedure does not preserve the phylogenetic structure within each population (i.e., randomized networks at later time points are not subnetworks of the randomized networks at earlier timepoints), for the sake of computational tractability. Duplication and amplification mutations are also ignored, owing to their rarity in these data (Tenaillon et al. 2016), and the evolution of new interactions is ignored due to a lack of data. Third, it is possible that gene essentiality evolves in the LTEE. Even though purifying selection on essential genes in the ancestral clone does not appear to be sufficient to cause selection for network resilience, it is possible that essential genes in the evolved clones make a greater contribution to network resilience than essential genes in the ancestral clone. Finally, the LTEE was specifically designed to minimize ecological complexity (Lenski et al. 1991). Given the significant correlation between network resilience and ecological complexity reported by Zitnik et al. (2019), it is possible that network resilience may often evolve under positive selection in nature, but not in the controlled and largely constant abiotic conditions of the LTEE.

Finally, there is an intriguing connection between network resilience and the deterministic mutation hypothesis for the evolution of sex (Kondrashov 1988; Azevedo et al. 2006). Loss-of-function mutations may have little effect on network integrity when they occur in a genome with high network resilience. By contrast, they may have catastrophic effects on network integrity when they occur in a genome with low network resilience. The deterministic mutation hypothesis states that synergistic epistasis between deleterious mutations—such as those that together cause network fragmentation—confers a selective advantage to sex. Near a critical threshold of network resilience, additional loss-of-function mutations are more likely to fragment biological networks, which could contribute to the synergistic epistasis required by the deterministic mutation hypothesis for the evolution of sex. Gene disruptions continue to accumulate over time in each LTEE population, suggesting that it might be worthwhile to test for such synergistic interactions at a later point (Elena and Lenski 1997), especially in the context of PPI network resilience.

## Supplementary Material

Supplementary data are available at *Genome Biology and Evolution* online.

## Supplementary Material

evab074_Supplementary_Data

## References

[evab074-B1] Azevedo RB , Lohaus R, Srinivasan S, Dang KK, Burch CL. 2006. Sexual reproduction selects for robustness and negative epistasis in artificial gene networks. Nature 440:87–90. 16511495 10.1038/nature04488

[evab074-B2] Ba ANN , et al 2019. High-resolution lineage tracking reveals travelling wave of adaptation in laboratory yeast. Nature 575:494–499. 31723263 10.1038/s41586-019-1749-3 PMC6938260

[evab074-B3] Callaway DS , Newman ME, Strogatz SH, Watts DJ. 2000. Network robustness and fragility: percolation on random graphs. Phys Rev Lett. 85:5468. 11136023 10.1103/PhysRevLett.85.5468

[evab074-B4] Cong Q , Anishchenko I, Ovchinnikov S, Baker D. 2019. Protein interaction networks revealed by proteome coevolution. Science 365:185–189. 31296772 10.1126/science.aaw6718 PMC6948103

[evab074-B5] Consuegra J , et al 2021. Insertion-sequence-mediated mutations both promote and constrain evolvability during a long-term experiment with bacteria. Nat Commun. 12:1–12. 33579917 10.1038/s41467-021-21210-7 PMC7881107

[evab074-B6] Couce A , et al 2017. Mutator genomes decay, despite sustained fitness gains, in a long-term experiment with bacteria. Proc Natl Acad Sci USA. 114:E9026–E9035. 29073099 10.1073/pnas.1705887114 PMC5664506

[evab074-B7] Elena SF , Lenski RE. 1997. Test of synergistic interactions among deleterious mutations in bacteria. Nature 390:395–398. 9389477 10.1038/37108

[evab074-B8] Good BH , McDonald MJ, Barrick JE, Lenski RE, Desai MM. 2017. The dynamics of molecular evolution over 60,000 generations. Nature 551:45–50. 29045390 10.1038/nature24287 PMC5788700

[evab074-B9] Grant NA , Maddamsetti R, Lenski RE. 2021. Maintenance of metabolic plasticity despite relaxed selection in a long-term evolution experiment with *Escherichia coli*. Am. Nat. Available from: 10.1086/714530. 34143718

[evab074-B10] Johnson MS , Martsul A, Kryazhimskiy S, Desai MM. 2019. Higher-fitness yeast genotypes are less robust to deleterious mutations. Science 366:490–493. 31649199 10.1126/science.aay4199 PMC7204892

[evab074-B11] Kafri M , Metzl-Raz E, Jona G, Barkai N. 2016. The cost of protein production. Cell Rep. 14:22–31. 26725116 10.1016/j.celrep.2015.12.015 PMC4709330

[evab074-B12] Kondrashov AS. 1988. Deleterious mutations and the evolution of sexual reproduction. Nature 336:435–440. 3057385 10.1038/336435a0

[evab074-B13] Leiby N , Marx CJ. 2014. Metabolic erosion primarily through mutation accumulation, and not tradeoffs, drives limited evolution of substrate specificity in *Escherichia coli*. PLoS Biol. 12:e1001789. 24558347 10.1371/journal.pbio.1001789 PMC3928024

[evab074-B14] Lenski RE. 2017. Experimental evolution and the dynamics of adaptation and genome evolution in microbial populations. ISME J. 11:2181–2194. 28509909 10.1038/ismej.2017.69 PMC5607360

[evab074-B15] Lenski RE , Rose MR, Simpson SC, Tadler SC. 1991. Long-term experimental evolution in *Escherichia coli*. I. Adaptation and divergence during 2,000 generations. Am Nat. 138:1315–1341.

[evab074-B16] Levy SF , et al 2015. Quantitative evolutionary dynamics using high-resolution lineage tracking. Nature 519:181–186. 25731169 10.1038/nature14279 PMC4426284

[evab074-B17] Łuksza M , Lässig M. 2014. A predictive fitness model for influenza. Nature 507:57–61. 24572367 10.1038/nature13087

[evab074-B18] Maddamsetti R. 2021. Universal constraints on protein evolution in the long-term evolution experiment with *Escherichia coli*. Genome Biol Evol. Available from: 10.1093/gbe/evab070. PMC8233687 33856016

[evab074-B19] Maddamsetti R , Grant NA. 2020a. Divergent evolution of mutation rates and biases in the long-term evolution experiment with *Escherichia coli*. Genome Biol Evol. 12:1591–1603. 32853353 10.1093/gbe/evaa178 PMC7523724

[evab074-B20] Maddamsetti R , Grant NA. 2020b. A simple test to infer mode of selection in metagenomics time series of evolving asexual populations. bioRxiv. Available from: 10.1101/2020.05.23.112508.

[evab074-B21] Maddamsetti R , et al 2017. Core genes evolve rapidly in the long-term evolution experiment with *Escherichia coli*. Genome Biol Evol. 9:1072–1083. 28379360 10.1093/gbe/evx064 PMC5406848

[evab074-B22] Mustonen V , Lässig M. 2010. Fitness flux and ubiquity of adaptive evolution. Proc Natl Acad Sci USA. 107:4248–4253. 20145113 10.1073/pnas.0907953107 PMC2840135

[evab074-B23] Ostrowski EA , Woods RJ, Lenski RE. 2008. The genetic basis of parallel and divergent phenotypic responses in evolving populations of *Escherichia coli*. Proc R Soc B Biol Sci. 275:277–284. 10.1098/rspb.2007.1244 PMC2593719 18029306

[evab074-B24] Papadopoulos D , et al 1999. Genomic evolution during a 10,000-generation experiment with bacteria. Proc Natl Acad Sci USA. 96:3807–3812. 10097119 10.1073/pnas.96.7.3807 PMC22376

[evab074-B25] Tenaillon O , et al 2016. Tempo and mode of genome evolution in a 50,000-generation experiment. Nature 536:165–170. 27479321 10.1038/nature18959 PMC4988878

[evab074-B26] Wiser MJ , Ribeck N, Lenski RE. 2013. Long-term dynamics of adaptation in asexual populations. Science 342:1364–1367. 24231808 10.1126/science.1243357

[evab074-B27] Woods R , Schneider D, Winkworth CL, Riley MA, Lenski RE. 2006. Tests of parallel molecular evolution in a long-term experiment with *Escherichia coli*. Proc Natl Acad Sci USA. 103:9107–9112. 16751270 10.1073/pnas.0602917103 PMC1482574

[evab074-B28] Woods RJ , et al 2011. Second-order selection for evolvability in a large *Escherichia coli* population. Science 331:1433–1436. 21415350 10.1126/science.1198914 PMC3176658

[evab074-B29] Zitnik M , Feldman MW, Leskovec J. 2019. Evolution of resilience in protein interactomes across the tree of life. Proc Natl Acad Sci USA. 116:4426–4433. 30765515 10.1073/pnas.1818013116 PMC6410798

