# Supplementary material for: Selection Maintains Protein Interactome Resilience in the Long-Term Evolution Experiment with Escherichia coli
Source: Genome Biol Evol. 2021 Apr 20;13(6):evab074. doi: 10.1093/gbe/evab074 (PMC8214405; doi:10.1093/gbe/evab074)

**Supplementary Figure S1**. Single-gene disruptions tend to increase the resilience of the REL606 PPI network. Each point represents the resilience of the REL606 PPI network after a single gene has been removed. The distribution of resilience effects after removing essential and nearly essential genes is shown on the left, and the distribution of resilience effects after removing non-essential genes is shown on the right. The dashed red line indicates the resilience of the original REL606 network. A) Analysis based on the *E. coli* PPI network published in Zitnik et al. (2019). B) Analysis based on the *E. coli* PPI network published in Cong et al. (2019).


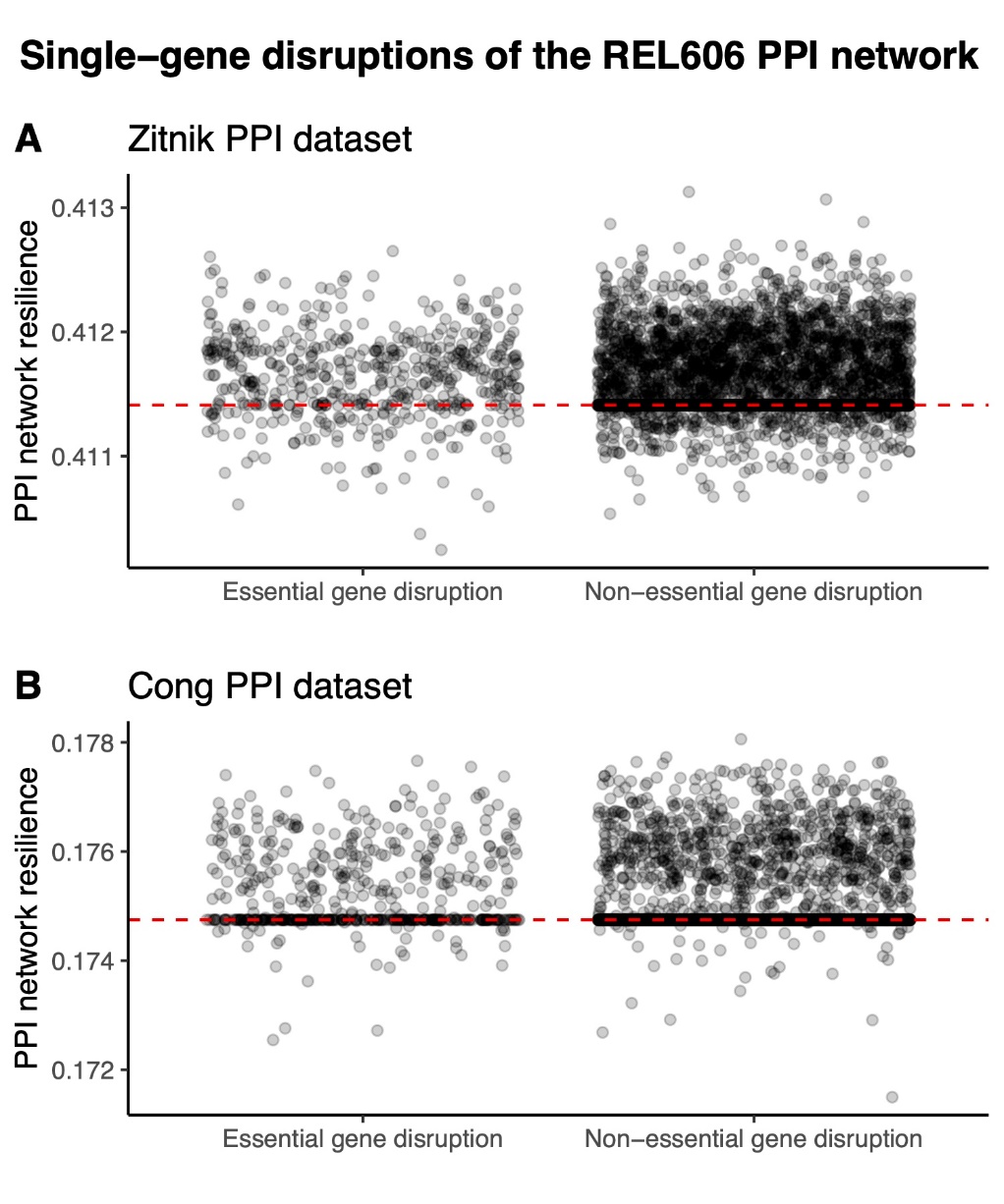


**Supplementary Figure S2.** The effects of single-gene disruptions on the resilience of 50,000 generation LTEE PPI networks. Each point represents the resilience of the PPI network after a single gene has been removed. In each subfigure, the top six populations have the ancestral point-mutation rate, while the bottom six populations evolved elevated point-mutation rates. Within each population-specific panel, the distribution of resilience effects after removing essential and nearly essential genes is shown on the left, and the distribution of resilience effects after removing non-essential genes is shown on the right. The dashed red line in each population-specific panel indicates the resilience of the original network for the given 50,000 generation LTEE clone. A) Analysis based on the *E. coli* PPI network published in Zitnik et al. (2019). B) Analysis based on the *E. coli* PPI network published in Cong et al. (2019).


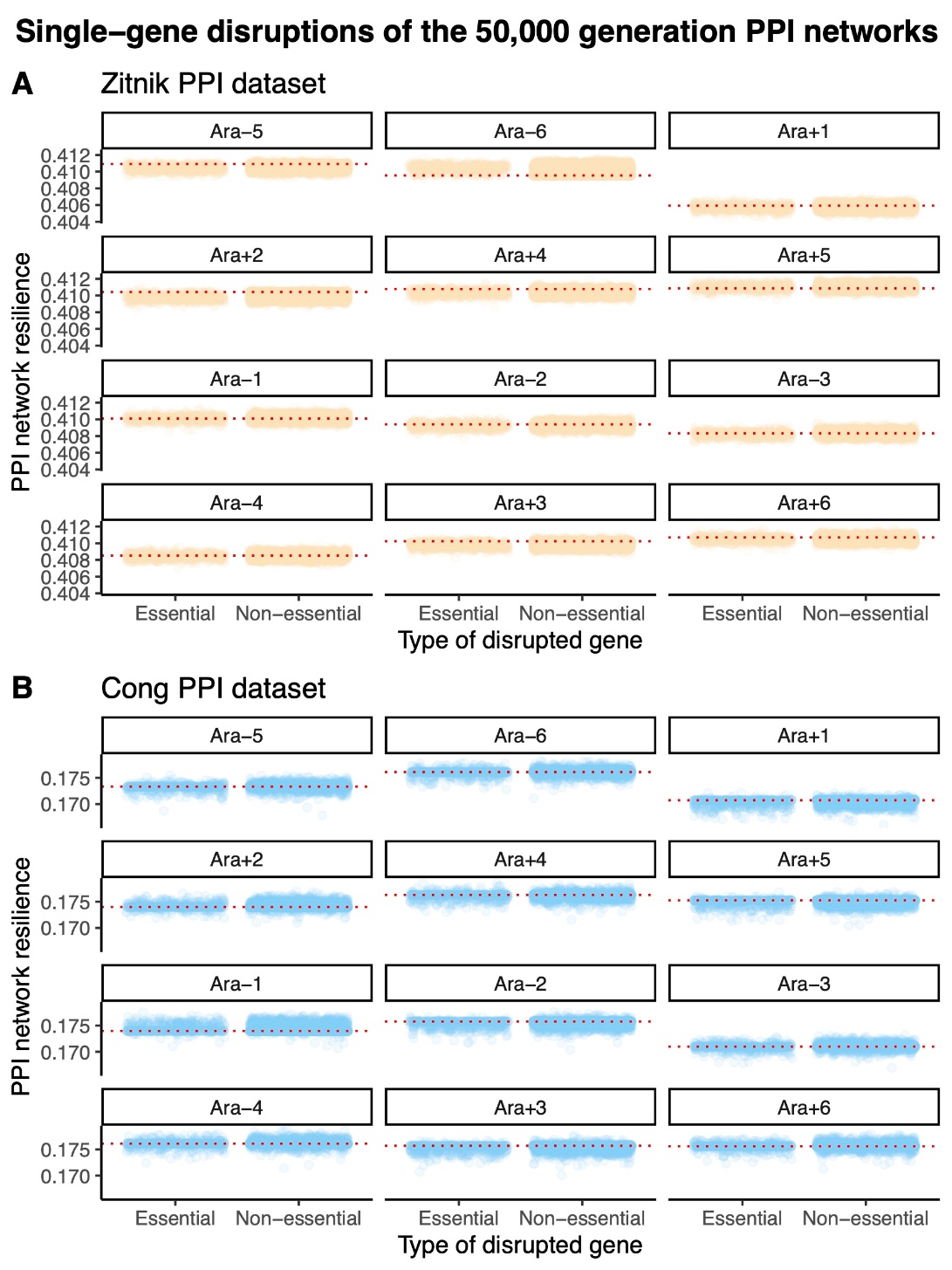

Supplement: evab074_Supplementary_Data [file evab074_supplementary_data.zip › 210135_Supplementary Figures.docx]
