# Supplementary material for: Selection Maintains Protein Interactome Resilience in the Long-Term Evolution Experiment with Escherichia coli
Source: Genome Biol Evol. 2021 Apr 20;13(6):evab074. doi: 10.1093/gbe/evab074 (PMC8214405; doi:10.1093/gbe/evab074)

SUPPLEMENTARY INFORMATION

Material and Methods

Datasets

I downloaded a table of nonsense SNPs, small indels, mobile element insertions, and large deletions affecting protein-coding regions of 264 genomes of LTEE clones isolated at 11 timepoints through 50,000 generations (Tenaillon, et al. 2016) using the web application at: https://barricklab.org/shiny/LTEE-Ecoli. The underlying data are also available at: https://github.com/barricklab/LTEE-Ecoli. Because these mutations disrupt protein reading frames, I use them as a proxy for loss-of-function mutations in the LTEE. For this reason, I call these types of mutations "gene disruptions", and call genes that are affected by these types of mutations "disrupted genes" for short. In the analyses restricted to 50,000 generation LTEE clones, I used single clones from each of the 12 populations (the LTEE 50,000 generation 'A' clones) to maximize statistical independence.

I used two different PPI datasets for *E. coli*. First, I used the curated PPI dataset published by Zitnik et al. (2019). This set of interactions corresponds to the interactome for species 511145 in their cross-species interactome dataset. For robustness, I also used a second *E. coli* protein interactome that has been published by Cong et al. (2019). This set of interactions includes those from protein structures in the Protein Data Bank (their Table S4), the Ecocyc database (their Table S5), yeast two-hybrid experiments (their Table S6), affinity purification and mass spectrometry (their Table S7), and those supported by known and high-confidence novel prediction coevolutionary information (their Tables S8 and S10, respectively). The Zitnik and Cong interactomes were filtered based on the genes they share with the *E. coli* B str. REL606 genome (the ancestral LTEE clone).

A list of essential and nearly essential genes in the LTEE ancestral clone REL606 was taken from Supplementary Table 1 of Couce et al. (2017), who identified these genes through transposon mutagenesis and sequencing. A comparison to the 57 genes with clear evidence of parallel evolution (i.e. two or more nonsynonymous mutations) in nonmutator lineages of the LTEE (Tenaillon, et al. 2016; Maddamsetti, et al. 2017) uses the data reported in supplementary table 2, Supplementary Material online of Tenaillon et al. (2016).

Network resilience analysis

The snap.py python module interface to the Stanford Network Analysis Platform (Leskovec and Sosič 2016) was used to generate a graph representing the ancestral REL606 interactome. Then, a protein interactome network was generated for each LTEE genome, by pruning the REL606 interactome of nodes (proteins) and edges (interactions) affected by gene disruptions in the given genome. Network resilience was calculated using the method described in Zitnik et al. (2019). In brief, resilience measures how well a network resists fragmentation into many small, isolated components as increasing fractions of nodes are randomly removed. Specifically, the entropy of the distribution of component sizes in the network is calculated, normalized by the logarithm of the total number of nodes in the original network. A fraction f of the nodes of the graph are randomly removed, ranging from 0.01 to 1.0, and the entropy of the network component distribution is calculated for each value of f. The entropy of the network component distribution increases monotonically as f increases. The area under the curve (AUC) of the mapping from f to the entropy of the network component distribution is calculated numerically, and network resilience is defined as 1 – AUC. This resilience calculation was conducted 100 times per genome. The average network resilience out of these 100 samples was used as the estimate for a genome's network resilience for a given simulation run. 100 simulations of this entire algorithm were conducted, such that network resilience was calculated 10,000 times for each genome. The trend over time in each population was then plotted, and a linear regression was fit to estimate the change in network resilience over time in each LTEE population. The y-intercept was fixed as the mean estimate for the resilience of the ancestral LTEE clone REL606 over all 100 simulation runs, so that the linear regression only fits one parameter (a slope representing network resilience over time). I then constructed two null distributions for comparison to the actual data as follows:

<u>Randomization over all genes in REL606.</u> For each of the 264 LTEE genomes (Tenaillon, et al. 2016), I constructed a corresponding randomized interactome network (Figure 1). The number of disrupted genes (i.e. genes removed from the ancestral interactome network) was fixed to the number of nonsense SNPs, small indels, mobile element insertions, and large deletions affecting protein-coding regions in the given LTEE genome. Then, a selection of genes in the ancestral REL606 genome was drawn at random. These genes were removed from the ancestral

interactome network, leaving a randomized network with the same number of disrupted genes as in the given evolved genome. The resilience of these randomized networks was calculated as described above.

Randomization over genes disrupted across LTEE populations. For each of the 264 LTEE genomes (Tenaillon, et al. 2016), I again constructed a corresponding randomized interactome network (Figure 1). As before, the number of disrupted genes was fixed to the number of nonsense SNPs, small indels, mobile element insertions, and large deletions affecting protein-coding regions in the given LTEE genome. In this case, however, a selection was drawn from the genes that were disrupted across all LTEE populations. I weighted the probability of selecting each gene based on the multiplicity of observed disruptions of that gene across LTEE populations. Specifically, the set of disrupted genes for each LTEE population was calculated, then the multiplicity of those genes across populations was calculated, such that a gene that was disrupted in 5 populations would be represented 5 times for sampling, while a gene that was never disrupted in any LTEE genomes would be omitted. The resilience of the resulting randomized network was calculated as described above.

The differences between the slopes for linear regressions for the actual data and the randomized data for each population were tabulated, and a Wilcoxon signed-rank test was used to test whether the paired difference between slopes, over all LTEE populations, was significantly greater than zero.

Single gene disruption analysis

Interactome resilience was calculated for the ancestral LTEE clone and the 12 50,000 generation LTEE 'A' clones, representing all 12 LTEE populations. For each clone, and for every protein-coding gene, the given protein was removed from the interactome network of the given clone, and interactome resilience was recalculated. If the given protein did not have any interactions in the network, then the resilience of the resulting network was set to the resilience of the original network for the clone. As before, each resilience calculation was conducted 100 times per genome, and the average network resilience out of these 100 samples was used to estimate the clone's network resilience. However, only one simulation of this entire algorithm

was conducted, such that network resilience was calculated 100 times for each of the ~4,000 networks corresponding to single-gene disruptions covering all protein-coding genes for each of the 13 genomes (ancestor and 12 clones) analyzed.

Supplementary Figure S1. Single-gene disruptions tend to increase the resilience of the REL606 PPI network. Each point represents the resilience of the REL606 PPI network after a single gene has been removed. The distribution of resilience effects after removing essential and nearly essential genes is shown on the left, and the distribution of resilience effects after removing non-essential genes is shown on the right. The dashed red line indicates the resilience of the original REL606 network. A) Analysis based on the *E. coli* PPI network published in Zitnik et al. (2019). B) Analysis based on the *E. coli* PPI network published in Cong et al. (2019).

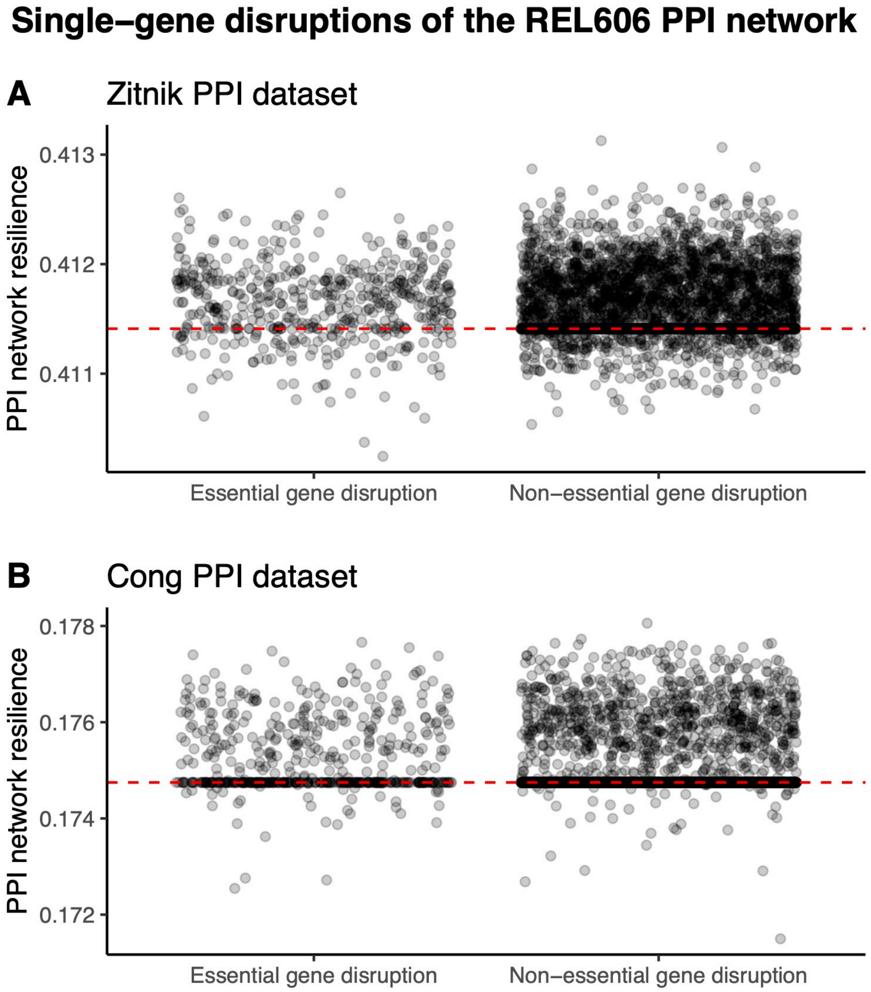

Supplementary Figure S2. The effects of single-gene disruptions on the resilience of 50,000 generation LTEE PPI networks. Each point represents the resilience of the PPI network after a single gene has been removed. The top six populations have the ancestral point-mutation rate, while the bottom six populations evolved elevated point-mutation rates. Within each population-specific panel, the distribution of resilience effects after removing essential and nearly essential genes is shown on the left, and the distribution of resilience effects after removing non-essential genes is shown on the right. The dashed red line in each population-specific panel indicates the resilience of the original network for the given 50,000 generation LTEE clone. A) Analysis based on the *E. coli* PPI network published in Zitnik et al. (2019). B) Analysis based on the *E. coli* PPI network published in Cong et al. (2019).

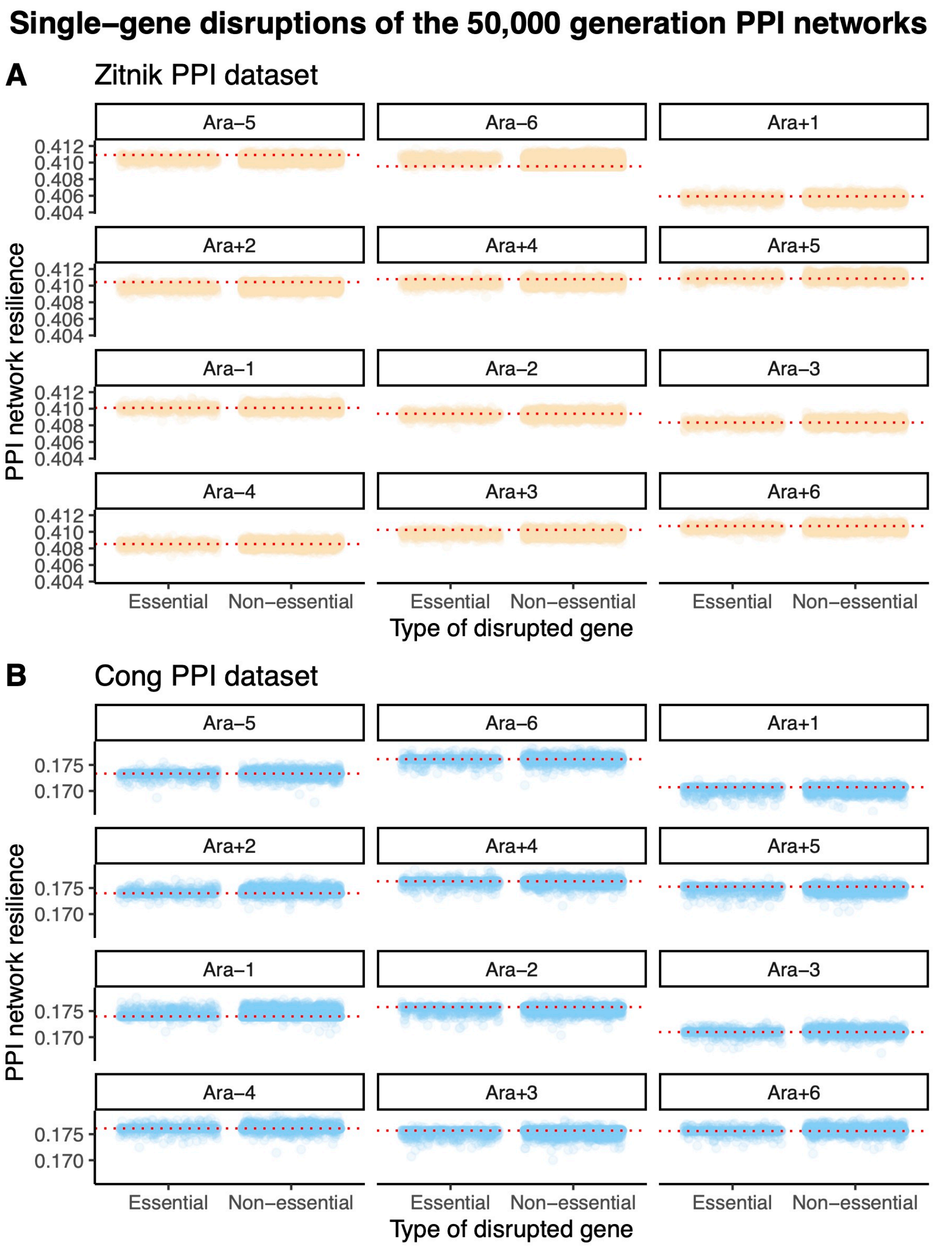

Supplement: evab074_Supplementary_Data [file evab074_supplementary_data.zip › Supplementary-Information.pdf]
